# Analysis of Time-Dependent Pharmacokinetics Using In Vitro–In Vivo Extrapolation and Physiologically Based Pharmacokinetic Modeling

**DOI:** 10.3390/pharmaceutics14122562

**Published:** 2022-11-22

**Authors:** Min-Chang Kim, Young-Joo Lee

**Affiliations:** 1 Department of Life and Nanopharmaceutical Sciences, Kyung Hee University, 26, Kyungheedae-ro, Dongdaemungu, Seoul 02453, Republic of Korea; 2 Division of Biopharmaceutics, College of Pharmacy, Kyung Hee University, Seoul 02447, Republic of Korea; 3 Department of Integrated Drug Development and Natural Products, Kyung Hee University, Seoul 02447, Republic of Korea

**Keywords:** auto-induction, time-dependent pharmacokinetics, SCR430, IVIVE, PBPK, isolated hepatocyte

## Abstract

SCR430, a sorafenib derivative, is an investigational drug exhibiting anti-tumor action. This study aimed to have a mechanistic understanding of SCR430’s time-dependent pharmacokinetics (TDPK) through an ex vivo study combined with an in vitro–in vivo extrapolation (IVIVE) and physiologically based pharmacokinetic (PBPK) modeling. A non-compartmental pharmacokinetic analysis was performed after intravenous SCR430 administration in female Sprague-Dawley rats for a control group (no treatment), a vehicle group (vehicle only, 14 days, PO), and a repeated-dosing group (SCR430, 30 mg/kg/day, 14 days, PO). In addition, hepatic uptake and metabolism modulation were investigated using isolated hepatocytes from each group of rats. The minimal PBPK model based on IVIVE was constructed to explain SCR430’s TDPK. Repeated SCR430 administration decreased the systemic exposure by 4.4-fold, which was explained by increased hepatic clearance (4.7-fold). The ex vivo study using isolated hepatocytes from each group suggested that the increased hepatic uptake (9.4-fold), not the metabolic activity, contributes to the increased hepatic clearance. The minimal PBPK modeling based on an ex vivo study could explain the decreased plasma levels after the repeated doses. The current study demonstrates the TDPK after repeated dosing by hepatic uptake induction, not hepatic metabolism, as well as the effectiveness of an ex vivo approach combined with IVIVE and PBPK modeling to investigate the TDPK.

## 1. Introduction

Time dependency in pharmacokinetics (PK) refers to time-dependent pharmacokinetic changes by multiple dosing. On occasion, the term “time-dependent” may also be used for chronopharmacokinetics that demonstrate pharmacokinetic changes by the actual time of administration. In this study, time-dependent pharmacokinetics (TDPK) was defined as the dosing period time-dependent pharmacokinetics [1].

A structural sorafenib analog, SCR430, is a new drug candidate that exhibits anti-tumor activity by inhibiting p-STAT3 and inducing SHP-1 [2,3] (ClinicalTrials.gov Identifier: NCT04733521). SCR430’s TDPK potential was suggested in its toxicokinetic study. The AUC and C_max_ decreased by 66% and 87%, respectively, in rats after repeated oral administration (30 mg/kg/day for 2 weeks).

The TDPK mechanism may vary. Metabolic enzyme auto-induction by repeated doses is a well-known mechanism, as reported in the cases of dexamethasone and rifampicin [4,5,6]. However, several biological factors besides metabolic enzymes govern drug elimination. For instance, the hepatic drug uptake and efflux transporters’ significance in the hepatic elimination process has been well documented, including safety, efficacy, and drug interactions [7,8,9]. Therefore, the auto-induction of such processes can also be the TDPK mechanism. Since hepatic elimination was expected to be the major elimination pathway of SCR430 by a preliminary PK study, this current study’s objectives were to investigate the underlying SCR430 TDPK mechanism by in vivo PK and hepatocyte ex vivo studies using in vitro–in vivo extrapolation (IVIVE) and physiologically based pharmacokinetic (PBPK) modeling [10,11].

## 2. Materials and Methods

### 2.1. Chemicals and Reagents

**Figure 1:**
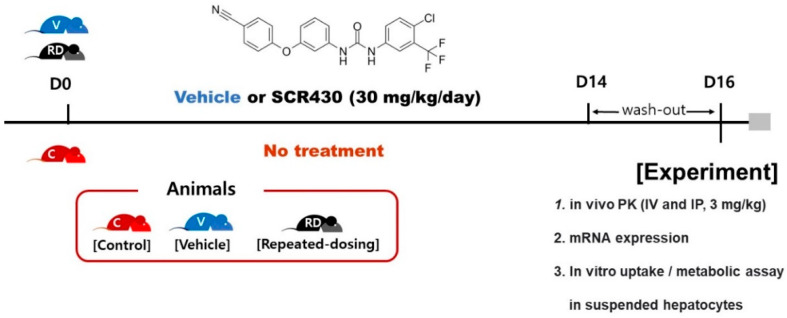
Schematic representation of the experimental procedure’s schedule. In this study, rats were divided into three groups. (1) Control group (red-colored, *n* = 6): the rats were not given a vehicle or drug; (2) Vehicle group (blue-colored, *n* = 6): the rats were given a vehicle once a day orally for 2 weeks; (3) Repeated-dosing group (black-colored, *n* = 6): the rats were given 30 mg/kg of the drug dissolved in the vehicle once a day orally for 2 weeks. After a washout period of two days, the SCR430 that was dissolved in the vehicle was administered intravenously or intraperitoneally at a dose of 3 mg/kg for each group.

SCR430 (Figure 1) was kindly supplied by RaND Biosciences Inc. (Seongnam, Republic of Korea). Sorafenib, silicone oil, mineral oil, propylene glycol, kolliphor^®^ HS 15, Williams’ medium E, and Hank’s balanced salts without calcium chloride and magnesium sulfate were purchased from Sigma-Aldrich, Inc. (St. Louis, MO, USA). Collagenase II was purchased from the Worthington Biochemical Corporation (Lakewood, NJ, USA). HPLC or MS-grade solvents used in the analysis were purchased from Sigma-Aldrich, Inc. (St. Louis, MO, USA).

### 2.2. Rat In Vivo PK Studies

Female Sprague-Dawley rats (8 weeks old, weighing between 190 and 205 g, Dahan Bio Link, Eumseong, Republic of Korea) were used for the in vivo PK studies. The female rats were used to evaluate urinary clearance by catheter insertion. The in vivo PK studies were conducted according to the schematic representation shown in Figure 1. Briefly, the rats were divided into three groups. The first group (control group) did not receive any treatment. The second group (vehicle group) and the third group (repeated-dosing group) were administered orally with a vehicle (50% Solutol HS15/50% polypropylene glycol 400 (1 mL/kg)) and 30 mg/kg of SCR430 dissolved in the vehicle once a day for two weeks, respectively. After the washout period of two days, the SCR430 that was dissolved in the vehicle was administered intravenously or intraperitoneally at a dose of 3 mg/kg for each group (*n* = 6/group). As time-dependent clearance (CL) was suggested in a previous study, the PK profile after the intravenous or intraperitoneal administration was investigated. Blood samples were collected in heparinized tubes based on a pre-designed schedule for up to 10 h. The blood samples were immediately separated into plasma and stored at −80 °C until analysis. Urine and bile samples were also collected to evaluate urinary and biliary excretions. The urine samples were collected for up to 10 h using urethral catheterization in each group (*n* =3). The bile samples were collected according to the designed schedule for up to 10 h using bile-duct cannulation from each group (*n* =3). The urine and bile samples were stored at −80 °C until analysis. All of the experimental procedures were reviewed and approved by the Committee on the Care and Use of Laboratory Animals, Kyung Hee University (KHSASP-19-440).

### 2.3. Non-Compartmental Analysis and Related PK Parameters

A pharmacokinetic analysis was performed by non-compartmental analysis using the PK-Solver^®^ program [12]. The tissue-to-plasma partition coefficients (*Kp*) of the liver and kidney were measured 10 h after the IV administration using Equation (1), considering the remaining blood within the tissues [13]. The blood-to-plasma ratio (*R_b_*) was determined in triplicate [14]. Briefly, the drug stock was spiked with fresh whole blood and plasma obtained from the control and repeated-dosing rats at a final concentration of 1 μM, and the samples were incubated at 37 °C for 1 h. Then, the blood samples were separated into plasma by centrifugation at 3000× *g* for 10 min at 4 °C. SCR430 and its metabolites in the plasma, bile, urine, and liver were analyzed using LC-MS/MS and UPLC-qToF-MS. The detailed procedures are described in Appendix A.

### 2.4. Determination of Fraction Unbound in Rat Plasma (f_u,p_), Hepatocyte Suspension (f_u,inc,hepa_), and Intrahepatocyte (f_u,hepa_)

Equilibrium dialysis (RED) assays were used to determine the free fraction of SCR430 in the rat plasma of the control and repeated-dosing groups and the hepatocyte suspension of the control group. An RED device (Thermo Fisher Scientific, Rockford, USA) was prepared according to the manufacturer’s guidelines. A total of 300 μL of plasma and dead hepatocyte suspension containing 5 μM SCR430 and 0.1% DMSO and 500 μL of phosphate-buffered saline (PBS) with solutol^®^ (0.01% *v*/*v* in PBS) were loaded into the sample (donor) chamber and buffer (receiver) chamber, respectively [15]. The samples were incubated at 37 °C on an orbital shaker at 300 rpm for 16 h. At the end of the incubation, the samples from both chambers were collected. Then, an equal volume of each matrix was added for matrix matching to improve the analytical efficiency. Finally, a liquid–liquid extraction (LLE) was performed on the samples using methyl tert-butyl ether and quantified by LC-MS/MS analysis. The *f_u,inc,hepa_* of the repeated-dosing group was assumed to be equal to that of the control group because of the non-specific binding of SCR430 in the hepatocyte suspension.

The *f_u,hepa_* was calculated from the reciprocal of the hepatocyte to the medium concentration ratio of the suspended hepatocyte isolated from the control and repeated-dosing groups at 4 °C by an oil-spin method to be described later under the assumption that the cellular binding is not temperature-dependent and that the active transport is entirely abolished on ice [16].

(1) fu,hepa=1AcellVcellCbuffer

where *V_cell_* is the rat hepatocyte volume (3.68 ± 1.37 μL/10^6^ cells) [17], *A_cell_* represents the amount of SCR430 in the hepatocytes, and *C_buffer_* is the SCR430 concentration in the buffer layer. The incubation proceeded for 120 min at 4 °C.

### 2.5. Assessment of Intrinsic Metabolic Clearance (CL_int,met_), Hepatic Uptake Clearance (PS_inf_), and Liver-to-Plasma Unbound Drug Concentration Ratio at Steady State (Kp_uu,ss_) Using Isolated Hepatocytes

Fresh rat hepatocytes were obtained from the control and repeated-dosing groups using the two-step collagenase liver perfusion method, described by Seglen et al. [18].

The intrinsic metabolic clearance (*CL_int,met_*) was extrapolated from the in vitro intrinsic metabolic clearance (*CL_int,met,vitro_*), and the *CL_int,met,vitro_* was determined by the conventional hepatocyte metabolic stability assay, which measures the parent drug depletion in the whole incubation medium [19]. Briefly, a drug stock (1 mM) dissolved in dimethyl sulfoxide and freshly isolated hepatocytes (1 × 10^6^ cells/mL) obtained from rats in the control, vehicle, and repeated-dosing groups were pre-incubated at 37 °C for 15 min. The metabolic reaction was initiated by adding 1 μL of the pre-incubated drug into 999 μL of the pre-incubated hepatocyte suspension in a buffer medium to achieve a final concentration of 1 μM containing 0.1% (*v*/*v*) dimethyl sulfoxide. At various time intervals (0, 5, 10, 20, 30, and 60 min), aliquots (50 μL) were transferred to 1 mL of ice-cold methyl tert-butyl ether solution. The extraction process and LC-MS/MS analysis were performed as described in Appendix A. Based on the predicted degradation half-life from the peak area ratio of the time profile data, the *CL_int,met,vitro_* (microliters per minute per 10^6^ cells) was calculated.

The *CL_int,met_* was determined using the following equation from *CL_int,met,vitro_*:

(2) $$CL_{int,\;met}=\frac{CL_{int,\;met,\;vitro}}{f_{u,\;inc,\;hepa\;}}\times pSF$$

The physiologic scaling factor for the rats (*pSF*) was 10^8^ million cells/g liver and 36 g liver/kg body weight [20].

The *PS_inf_*, the sum of *PS_inf,act_* + *PS_diff_*, was extrapolated from the in vitro hepatocyte uptake clearance (*PS_inf,hep_*), and the *PS_inf,hep_* was determined by an integration plot analysis by an oil-spin uptake assay using the suspended hepatocytes isolated for the control and repeated-dosing groups using the following Equations (3) and (4) [21,22].

The uptake assay was performed at 37 °C (for the active and passive uptakes) and at 4 °C (on ice for the passive uptake) to discriminate between the active and passive processes. The hepatic uptake was initiated by adding buffers containing the drug (2 μM in 0.2% DMSO) to the suspended hepatocytes in the buffer at an equal volume, resulting in the final concentration of the drug (1 μM in 0.1% DMSO) and a hepatocyte concentration of 0.5 × 10^6^ cells/mL. After 10, 45, 60, 90, and 120 s, aliquots (100 μL) were removed from each tube and transferred to a silicon layer tube that consisted of two layers; the upper layer included 100 µL of oil mixture (silicon oil (81.7)/mineral oil (18.3), *v*/*v*, with a density of 1.015 g/mL) and the bottom layer included 50 µL of 1 M sucrose. Then, centrifugation was performed at 7000× *g* for 10 s to distinguish between the hepatocytes and the medium buffer. After the centrifugation, the upper- and lower-layer samples were separated and kept in a −80 °C deep freezer until further analysis.

The *PS_inf,hep_* was calculated from the slope of the integration plot and used for the extrapolation.

(3) $$\frac{X_{hep\;\left(t\right)}}{C_{buff\left(t\right)}}=PS_{\;inf,hep}\times \frac{AUC_{\left(0-t\right),\;buff}}{C_{buff\left(t\right)}}+V_{0}$$

(4) $$PS_{inf}=\frac{PS_{\;inf,hep}}{f_{u,\;buff\;}}\times pSF$$

where *X_hep(t)_*_,_ *C_buff(t)_*, *AUC_(_*_0*−t),buff*_, *f_u,buff_*, and *V*_0_ represent the amount of the drug that was taken into the hepatocytes, the concentration of the drug in the buffer layer after centrifugation, the area under the drug concentration–time curve in the buffer layer, the unbound fraction of the drug in the buffer layer, which was assumed to be 1, and the initial volume of distribution at the initial stage, respectively. The *X_hep(t)_*/*C_buff(t)_* value was plotted against the *AUC_(_*_0*−t),buff*_/*C_buff(t)_* value, and the *PS_inf,hep_* (µL/min/10^6^ cells) was estimated from the initial slope obtained from the linear plot.

The *Kp_uu,ss_* was determined using rat hepatocytes isolated from the control and repeated-dosing groups. A temperature-dependent steady-state uptake method by oil-spin uptake assay with 4% rat serum at 37 °C and 4 °C was used [16,22].

The *Kp_uu,ss_* was calculated using the following Equation (5):

(5) $$Kp_{uu,\;ss\;}=\frac{C_{u,\;hep,ss\;\left(37\;{}^{\circ}C\right)}}{C_{u,\;media,ss\;\left(37\;{}^{\circ}C\right)}}=\frac{C_{hep,ss\;\left(37\;{}^{\circ}C\right)}\times f_{u,hepa}}{C_{media,ss\;\left(37\;{}^{\circ}C\right)}\times f_{u,media}}=\frac{Kp_{\left(37\;{}^{\circ}C\right)}}{Kp_{\left(4\;{}^{\circ}C\right)}}$$

The *Kp* was determined by the ratio of the hepatocyte concentration and the medium concentration at 20 min (37 °C) and 120 min (4 °C).

### 2.6. Calculation of In Vivo Overall Intrinsic Clearance (CL_int,all_) and Overall Hepatic Clearance (CL_h_)

An IVIVE based on extended clearance concepts was applied to predict the *CL_int,all_* [11].

The *CL_int,all_* was defined as a function of the intrinsic metabolic CL (*CL_int,met_*), the hepatic diffusional passive (*PS_diff_*) and hepatic active uptakes (*PS_inf,act_*), and the efflux (*PS_eff,act_*) permeability. Biliary efflux was not counted because SCR430 was detected at a trace level in the bile.

(6) $$CL_{int,\;all\;}=CL_{int,\;met\;}\times \frac{\left(PS_{inf,\;act}+PS_{diff}\right)}{\left(PS_{eff,\;act}+PS_{diff}+CL_{int,\;met\;}\right)}$$

The *CL_int,all_* was calculated in three ways [16,22,23].

Method 1: Determination of the *CL_int,all_* by assuming the intrinsic metabolic clearance dependent (*CL_int,met_*).

Method 1 assumed that the active transport was negligible (*PS_inf,act_* and *PS_eff,act_* « *PS_diff_*) and that the hepatic diffusional passive permeability greatly exceeded the intrinsic metabolic clearance (*PS_diff_* » *CL_int,met_*) in Equation (6) [22].

(7) $$CL_{int,\;all\;}≅CL_{int,\;met\;}$$

Method 2: Determination of the *CL_int,all_* by assuming the hepatic uptake clearance (*PS_inf_*).

If the *CL_int,met_* is much greater than the overall total efflux clearance (*PS_eff,act_* + *PS_diff_*) in Equation (6), the *CL_int,all_* is dominated by the overall hepatic uptake clearance (*PS_inf_* = *PS_inf,act_* + *PS_diff_*) as the rate-limiting step [16,24].

(8) $$CL_{int,\;all\;}≅PS_{inf}$$

Method 3: Determination of the *CL_int,all_* based on the liver-to-plasma unbound drug concentration ratio at a steady state (*Kp_uu,ss_*).

The right part of Equation (6) for the *CL_int,all_*, (*PS_inf,act_* + *PS_diff_*)/(*PS_eff,act_* + *PS_diff_* + *CL_int,met_*) corresponds to the *Kp_uu,ss_*, regardless of the assuming rate-limiting step [22,25,26].

(9) $$CL_{int,\;all\;}=CL_{int,\;met}\times Kp_{uu,ss}$$

### 2.7. Determination of Fraction Unbound in Rat Plasma (f_u,p_), Hepatocyte Suspension (f_u,inc,hepa_), and Intrahepatocyte (f_u,hepa_)

The hepatic clearance (*CL_h_*) was predicted from the following equations based on a dispersion model using the *CL_int,all_* derived from the above three methods (Method 1, 2, and 3) [24]:

(10) $$CL_{h}=Q_{h\;}\times (1-F_{h})\times R_{b}$$

(11) $$F_{h\;}=\frac{4a}{{\left(1+a\right)}^{2}\times \exp\left(\frac{a-1}{2D_{N}}\right)-{\left(1-a\right)}^{2}\times \exp\left(-\frac{a+1}{2D_{N}}\right)}$$

(12) $$a={\left(1+4R_{N}\times D_{N}\right)}^{\frac{1}{2}}$$

(13) $$R_{N}=f_{u,blood}\times \frac{CL_{int,\;all\;}}{Q_{h\;}}$$

The *D_N_*, dispersion number, was set to 0.17 [27], and *Q_h_* was 3.69 L/h/kg [28].

### 2.8. Accuracy, Precision, and Bias Assessments of the Model Predictions

The accuracy of the estimated *CL_h_* was calculated by the *fold error* method, based on whether the ratio of the model-predicted values fell within 3-fold of the observed in vivo *CL_h_* values in the rats [16].

(14) $$fold\;error=10^{\log_{10}\frac{predicted\;CL_{h}}{observed\;CL_{h}\;}}$$

The following equation uses the *absolute average fold error* (*AAFE*) to measure the bias:

(15) $$AAFE=10^{\frac{1}{n}\sum^{\;}\log\left(\frac{predicted\;CL_{h}}{observed\;CL_{h}\;}\right)}$$

### 2.9. The Minimal PBPK (mPBPK) Model Construction for the Control and Repeated-Dosing Groups

The liver was considered the major elimination pathway, considering low biliary and renal clearance data. Thus, the mPBPK containing the liver and a single-adjustment compartment was constructed to predict the plasma and liver concentrations of the control and repeated-dosing groups (Figure 7). To emulate the dispersion model, the liver compartment was divided into five units, which were composed of extracellular and hepatocellular units linked by hepatic blood flow [29]. The mass-balanced differential equations for the mPBPK model referring to the liver are shown in Appendix A. The kinetic input parameters for the mPBPK modeling of the control and repeated-dosing groups are summarized in Table 3. The *f_u,hepa_* values of the control and repeated-dosing groups were assumed to be equal to the *f_u,hepa_* of the control and repeated-dosing groups. The *f_u,b_* was calculated from the *f_u,p_*, and the *R_b_*.

The mPBPK model was coded using Berkeley Madonna (version 8.3.23, University of California at Berkeley, CA, USA). The performance of the constructed PBPK model was assessed using the determination coefficient (R^2^) and the mean absolute percentage error.

### 2.10. Sensitivity Analysis

Using Berkeley Madonna, a local sensitivity analysis was performed to determine the parameters that have the most crucial influence on the plasma or liver AUC for SCR430. The differences in the AUC were estimated after each parameter was increased by 10%. Equation (16) was used to compute the normalized sensitivity coefficient (NSC) [30,31,32,33], where *r* and *r*′ represent the initial value and the modified value of the plasma or liver AUC by the 10% increase in the interest parameter (e.g., *f_u,hepa_*, *PS_inf,act_*), respectively. Additionally, p and p′ represent the initial value and the 10% increased value of the interest parameter, respectively. The relative impact of each parameter on the plasma and liver AUC was categorized as follows: low: |NSC| < 0.2; medium: 0.2 ≤ |NSC| < 0.5; high: 0.5 ≤ |NSC| [34].

(16) $$\mathrm{NSC}\;=\frac{r^{\prime }-r}{r}\times \frac{p}{p^{\prime }-p}$$

### 2.11. Comparison of the Model Predictions Using Monte Carlo Simulation and Observed Data of Plasma and Liver Concentrations

The Monte Carlo simulation (*n* = 1000) was employed to assess the impact of uncertainties in the parameter values, including the hepatic uptake or metabolic process and the unbound fraction in the liver (*f_u,hepa_*) on the plasma and liver concentrations in the control and repeated-dosing groups. Lognormal distributions were assumed for the model parameters, including *f_u,hepa_*, *PS_inf,act_*, *PS_diff_*, and *CL_int,met_*.

The probabilistic distributions of the lognormally distributed parameter values were defined by their mean values and the variability of the in vitro data derived from the uptake, metabolic stability, and binding assays using the rat hepatocytes. First, the skewed parameters with a lognormal distribution were transformed into a normally distributed variable ω, with the mean (*μ_x_*) and the standard deviation (σ*_x_*) using the equation shown as follows [35]:

(17) $$\mu_{\omega }=\ln(\mu_{x}^{\;2}\;/\;\sqrt{\sigma_{x}^{\;2}+\mu_{x}^{\;2}}$$

(18) σω=ln(1+σx 2μx 2)

(19) $$\mathrm{Lognormal}\;\left(\mu_{x},\;\sigma_{x}\right)=\exp(\mathrm{Normal}\;\left(\mu_{\omega },\;\sigma_{\omega }\right)$$

where *μ_x_* and σ*_x_* are the mean and the standard deviation for the lognormal distribution of the parameters derived from the in vitro data, respectively; moreover, *μ*_ω_ and σ_ω_ represent the mean and the standard deviation following the conversion to a normal distribution, respectively [36].

The Monte Carlo simulations were implemented within Berkeley Madonna as described by Li et al. [35]. Intravenous doses of 3 mg/kg of the SCR430 were simulated for the plasma and liver concentrations of the control and repeated-dosing groups, respectively. Excel and GraphPad Prism 6.0 (GraphPad Software Inc., San Diego, CA, USA) were used to calculate the 5th, 20th, 35th, 50th, 65th, 80th, and 95th percentiles of the liver and plasma concentrations in the control and repeated-dosing groups.

### 2.12. Gene Expression Analysis Using Quantitative PCR (qPCR)

The Rat pregnane X receptor (rPXR), the rat farnesoid X receptor (rFXR), the rat Cytochrome P450 3a2 (rCyp3a2), the rat organic cation transporter1 (rOct1), and the rat organic anion transporting polypeptides (rOatp1a1, rOatp1a4, and rOatp1b2) were chosen as the representative genes related to hepatic auto-induction or elimination. The mRNA expression of the genes was monitored using real-time qPCR in the liver that was extirpated from the rats (*n* = 3) and an in vivo PK study was completed in the control and repeated-dosing groups. According to the manufacturer’s instructions, the total RNA was extracted from the liver using RNAiso (Takara, Tokyo, Japan) and used for the qPCR [37]. The primer sequences and PCR conditions for the qPCR are summarized in Appendix B, Table A1. The result of integrating the standard deviation of the ΔΔCT value was used to calculate the fold difference [38].

## 3. Results

### 3.1. TDPK of SCR430

**Figure 2:**
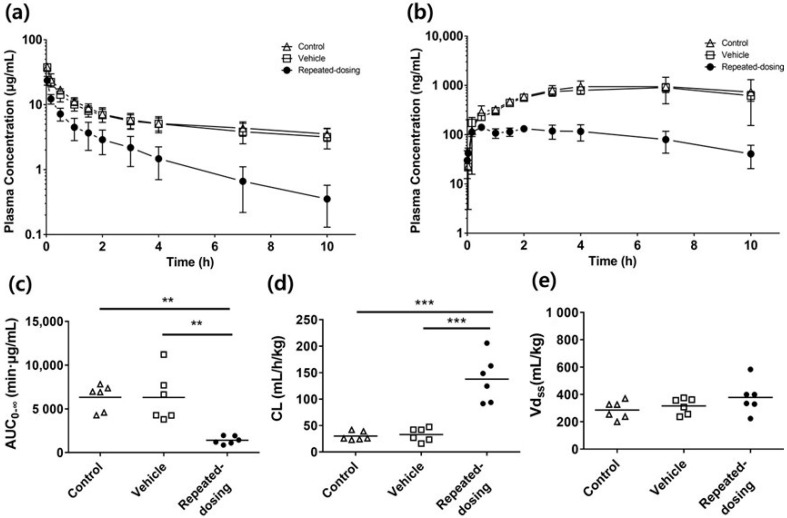
(**a**,**b**) Mean plasma concentration–time curve of SCR430 after IV (**a**) or IP (**b**) administration at a dose of 3 mg kg^−1^ (△, control (without a dosing vehicle or SCR430) group; □, vehicle (vehicle only, 14 days, PO) group; ●, repeated-dosing (SCR430 30 mg/kg, 14 days, PO) group (*n* = 6). (**c**–**e**) Comparison of the control, vehicle, and repeated-dosing groups’ pharmacokinetic parameters. The AUC from time 0 h to infinity (AUC_0–∞_) (**c**), the total plasma clearance (CL) (**d**), and the steady-state volume of distribution (Vd_ss_) (**e**) were compared. A one-way ANOVA statistical analysis was used for the statistical analysis, followed by a Turkey’s post-hoc test, where appropriate. The stars indicate statistical significances, ** *p* < 0.01, *** *p* < 0.001.

**Figure 3:**
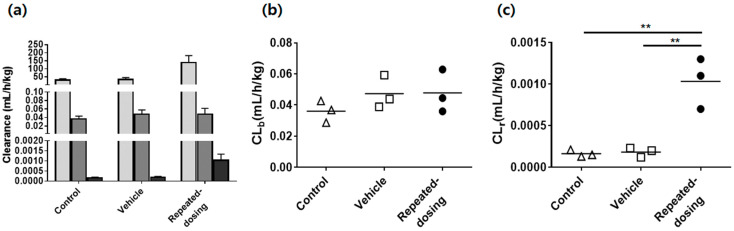
(**a**) Comparison of the total (light gray, *n* = 6), biliary (dark gray, *n* = 3), and urinary (black, *n* = 3) SCR430 clearance after IV SCR430 bolus administration (3 mpk) in the control, vehicle, and repeated-dosing groups. The biliary clearance (CL_b_) (**b**) and urinary clearance (CL_r_) (**c**) were also compared. A one-way ANOVA statistical analysis was used for the statistical analysis, followed by a Turkey’s post-hoc test, where appropriate. The stars indicate statistical significances, ** *p* < 0.01.

The SCR430’s mean plasma concentration–time curves after the intravenous bolus administration dosing at 3 mg/kg in the control, vehicle, and repeated-dosing groups indicated SCR430’s TDPK nature (Figure 2a). The repeated-dosing group showed lower plasma concentrations with a more rapid elimination phase than the control and vehicle groups. In contrast, the control and vehicle groups’ plasma profiles were similar. The PK parameters that were estimated by the non-compartmental analysis indicated the SCR430’s time-dependent CL (Figure 2c–e, and Table 1). The repeated-dosing group’s AUC_0–∞_ and CL showed a statistically significant difference in the AUC_0–∞_ and CL compared to the control and vehicle groups, whereas the volume of distribution at the steady state (Vd_ss_) was similar. An approximately 4-fold decrease in the AUC_0–∞_ (*p* = 0. 0013) and an increase in the CL (*p* < 0.001)) were observed. In contrast, the vehicle group’s AUC_0–∞_, CL, and Vd_ss_ were not statistically different from those of the control group (*p* > 0.05). Additionally, similar results were observed after an IP administration, where TDPK was more clearly identified after the IP administration (Figure 2b). The plasma concentration of the repeated-dosing group was much lower than that of the control and vehicle groups, and an 8-fold decrease in the AUC_IP,0–10_ was observed in the repeated-dosing group compared to the control group (*p* < 0.001). The biliary and renal clearances were negligible and less than 0.1% compared to the total clearance in all of the groups (Figure 3a, Table 1). SCR430’s biliary clearance did not show any statistical differences between the groups (Figure 3b). However, the urinary clearance was higher in the repeated-dosing group (Figure 3c). The *Kp* of the liver in all of the groups presented no significant differences. The *Kp* of the kidney was higher in the repeated-dosing group than in the control and vehicle groups (Table 1). The *R_b_* values were calculated as 3.95, 4.58, and 4.53 in the control, vehicle, and repeated-dosing groups, respectively. There were no significant differences in the groups (Table 1).

### 3.2. Metabolite Identification and Quantification of SCR430

**Figure 4:**
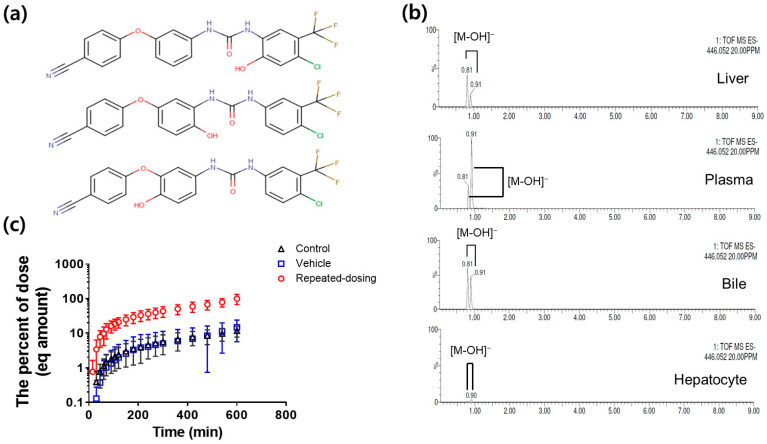
(**a**) Three candidate metabolites of the SCR430 aromatic hydroxyl site via CYP-mediated metabolism predicted by metabolite profiling. (**b**) Representative UPLC-qToF-MS chromatograms for the two hydroxylated SCR430 metabolite types in the liver tissue, plasma, bile, and hepatocytes of the repeated-dosing group. (**c**) The equivalent cumulative percentage–time profile of the dose recovered as an aromatic hydroxylated metabolite in the bile after IV administration among the control, vehicle, and repeated-dosing groups. (Data = mean ± standard deviation (SD), *n* = 3).

SCR430’s dominant metabolites were predicted as hydroxyl metabolites by in silico metabolite profiling using GLORY [41]. From three candidate metabolites for the SCR430 site (Figure 4a), two structurally different hydroxylated metabolite types at *m*/*z* 446.052 [M-OH]^−^ were confirmed using UPLC-qToF-MS in the plasma, liver tissue, bile acid, and hepatocyte samples (Figure 4b). The identified metabolites were not practically analyzed in urine samples. Due to chemical standard unavailability for the identified metabolites, the equivalent hydroxylated metabolite concentration was predicted using LC-MS/MS on the assumption that the linearity of the metabolite MS response and the concentration is equivalent to the parent drug. The equivalent hydroxylated metabolite amounts excreted in the bile for up to 10 h from each group were converted into percent of dose. Approximately ten times more metabolites were generated in the repeated-dosing group than in the control and vehicle groups (Figure 4c).

### 3.3. Low Fraction Unbound of SCR430 in the Plasma and Liver

The *f_u,p_*, *f_u,inc,hepa_*, and *f_u,hepa_* values were low and determined to be 0.0078, 0.032, and 0.004 for the control group, respectively (Table 1). These low values of fraction unbound may be due to SCR430’s high lipophilicity (clogP = 6.15, Appendix B, Table A2). The *f_u,p_* and *f_u,inc,hepa_* values predicted by an in silico prediction based on physicochemical properties (Appendix B, Table A2) was 0.0066 [42] and 0.024 [39], similar to the observed values, which were 0.0078 and 0.032.

Interestingly, the repeated-dosing group’s *f_u,hepa_* value was 2.5-fold higher than the control group’s value, whereas the control group’s *f_u,p_* was similar to the vehicle and repeated-dosing groups’ values. Although the reason was yet unclear, this difference clearly explained the liver concentration difference in the repeated-dosing group, which will be described later.

### 3.4. Similar CL_int,met_ between the Control, Vehicle, and Repeated-Dosing Groups

The difference in the *CL_int,met_* by the hepatic metabolism between the three groups was assessed by a conventional metabolic stability assay using the suspended rat hepatocytes.

**Figure 5:**
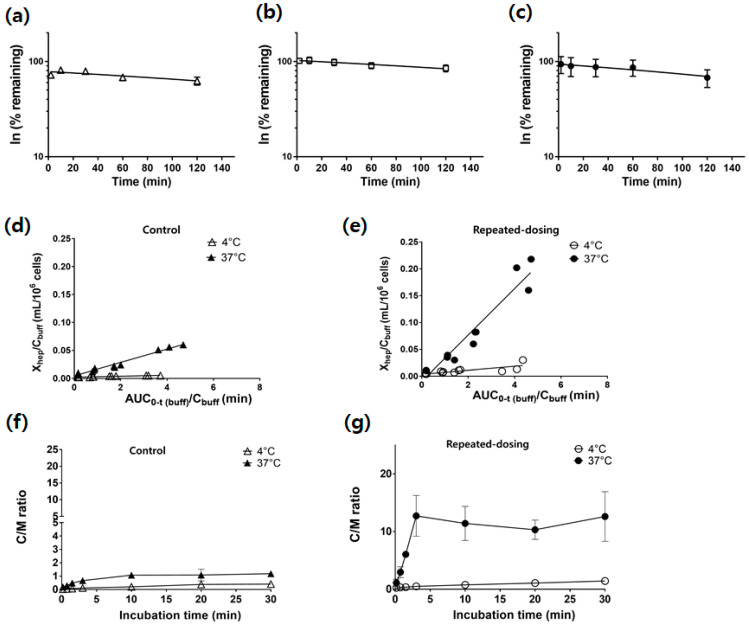
(**a**–**c**) The degradation rate of SCR430 in the suspended hepatocytes from the three groups of rats: control (**a**), vehicle (**b**), and repeated-dosing (**c**) groups. (**d**,**e**) Integration plots for the hepatic uptake clearance of SCR430 in the control (**d**) and repeated-dosing (**e**) groups at the condition of 4 °C (open) and 37 °C (close). The amount of SCR430 within the hepatocytes divided by the concentration (t) in the buffer was plotted against the AUC_0–∞_ (buff) divided by the concentration (t) in the buffer. The solid lines represent the linear regression line for the control and repeated-dosing groups. (**f**,**g**) The *Kp* (C/M ratio) of SCR430 was assessed at 4 °C (open) and 37 °C (close) to determine the *Kp_uu,ss_* in the control (**f**) and repeated-dosing groups (**g**) (Data = mean ± SD).

A relatively slow depletion of SCR430 was observed with monophasic kinetics. The *CL_int,met_* of the control, vehicle, and repeated-dosing group was 1.32, 1.21, and 1.69 µL/min/10^6^ cells, respectively (Figure 5a–c). No significant difference in the *CL_int,met_* was observed between the groups (Table 2).

### 3.5. Increased PS_inf,hep_ and Kp_uu,ss_ in the Repeated-Dosing Group

The time-dependent *PS_inf,hep_* and *Kp_uu,ss_* potentials were investigated in the suspended hepatocytes isolated from the control and repeated-dosing groups’ livers (Figure 5d–g). The *PS_inf,hep_* calculated from the initial uptake clearance by an integration plot was significantly increased in the repeated-dosing group compared to the control group (*p* = 0.0263, Table 2). The control and repeated-dosing groups’ *Kp_uu,ss_* values were determined by a temperature method at 4 °C and 37 °C and were 0.11 and 1.7, respectively (Table 2). An approximately 15-fold *Kp_uu,ss_* value increase in the repeated-dosing group was observed compared to the control group (*p* < 0.001).

### 3.6. Comparison of CL_h_ Determined by Three IVIVE Methods and In Vivo Clearance in the Control and Repeated-Dosing Groups

**Figure 6:**
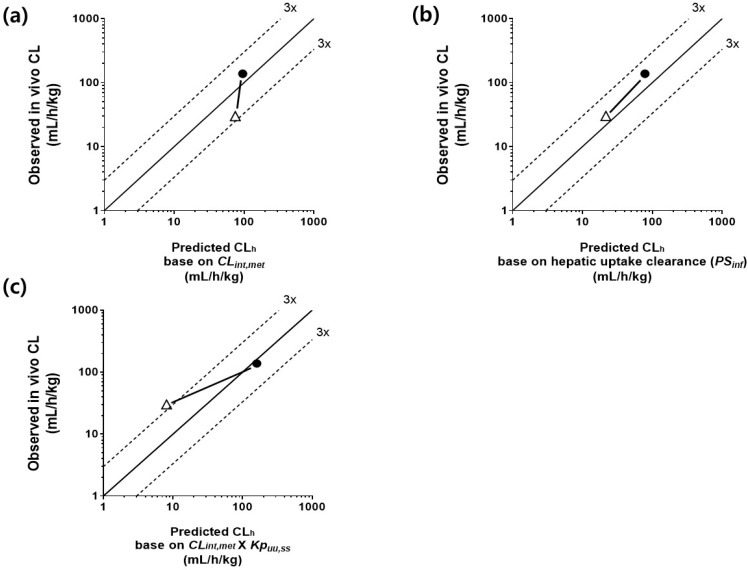
In vitro predicted hepatic clearance correlation with the observed hepatic clearance in the control (open triangle) and repeated-dosing (closed circle) groups. (**a**) Method 1: Predicted hepatic clearance based on the intrinsic metabolic clearance (*CL_int,met_*). (**b**) Method 2: Predicted hepatic clearance based on the hepatic uptake clearance (*PS_inf_*). (**c**) Method 3: Predicted hepatic clearance based on the corrected *CL_int,met_* by multiplying with *Kp_uu,ss_*. The solid lines represent the line of unity, which is the best fit, and the dashed lines represent the 3-fold error from the unity.

The CL_h_ was predicted by the following three IVIVE methods: (1) intrinsic metabolic clearance, (2) hepatic uptake clearance, and (3) liver-to-plasma unbound drug concentration ratio at steady state. They were determined in the control and repeated-dosing groups and used for the CL_h_ prediction (Table 2). The observed total in vivo CL was compared with the predicted CL_h_ by the three IVIVE methods (Figure 6), as SCR430’s renal clearance was negligible. The CL_h_ obtained by Method 2 were clearly explained by the observed in vivo CL within a 3-fold (Table 2) and were centered along the unity line (Figure 6b). However, Method 1 failed to explain the difference in the in vivo CL between the control and repeated-dosing groups (Figure 6a). Furthermore, there was no statistical difference in the predicted CL_h_ between the control and the repeated-dosing groups. Method 3 showed a predicted value that was slightly overestimated over 3-fold for the control group, and an increased CL in the repeated-dosing group was partially predicted (Figure 6c). Considering the uncertainty caused by the *f_u,inc,hepa_* (0.032) in calculating the *CL_int,met_* [43], the other values of *f_u,inc,hepa_* (0.024, value from the in silico prediction [39], 0.088, extrapolation from *f_u,p_* [40], and unity) were also used in Methods 1 and 3, but similar results were observed. Method 1 still did not reflect the change in the in vivo CL in the repeated-dosing group. In the case of Method 3, the absolute difference between the predicted and observed values was still larger than Method 2, although the change in the repeated-dosing group was partially reflected.

When the data were analyzed by the *AAFE* method, the 3-fold error range was between 0.37 and 2.8. The *AAFE* value for the CL_h_ calculated by Method 1 in the control group was 2.4, indicating a disagreement between prediction and observation. The *AAFE* values for the CL_h_ calculated by Methods 2 and 3 were 0.73 and 0.26, respectively, for the control group and 0.53 and 0.99, respectively, for the repeated-dosing group. These results suggested that the hepatic uptake was a more critical factor in explaining the SCR430 TDPK.

### 3.7. An mPBPK Model Could Predict the Differences in Plasma and Liver Concentrations in Control and Repeated-Dosing Groups

**Figure 7:**
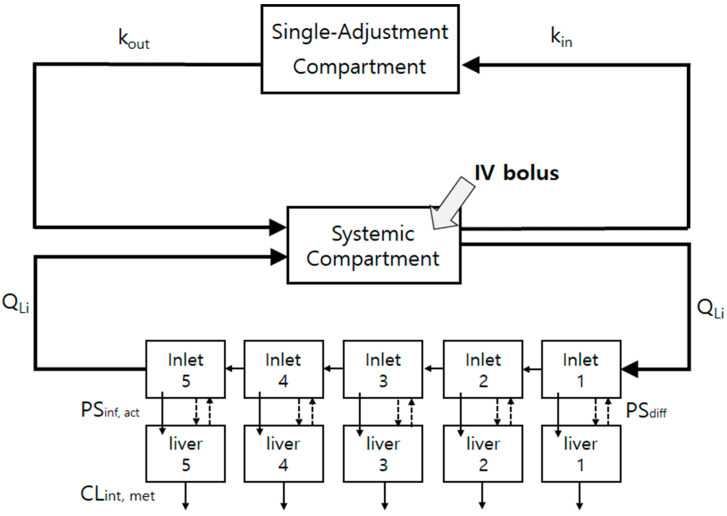
Schematic diagram of the mPBPK model with a single-adjustment compartment was designed to predict the plasma and liver concentrations in the control and repeated-dosing groups. The liver unit was composed of five units to mimic the dispersion model. The dotted, dashed line between the inlet and liver units is the passive diffusion clearance (*PS_diff_*), and the solid downward line is the active transporter-mediated uptake (*PS_inf,act_*) clearance. The solid downward line at the bottom of the liver units is the metabolic clearance (*CL_int,met_*).

An mPBPK model has been developed to elucidate how changes in the hepatic uptake by auto-induction affect the overall plasma and liver concentrations (Figure 7). The volumes of the single-adjustment and systemic compartments and the rate constant to the single-adjustment compartment (k_in_) and from (k_out_) were fitted to the actual plasma concentration profile. The *PS_inf_* was subdivided into passive diffusion clearance (*PS_diff_*) and active hepatic uptake clearance (*PS_inf,act_*) for the liver modeling. The *PS_inf,act_* and *PS_diff_* were calculated using IVIVE Method 2, and the empirical scaling factor (1.6) was multiplied to match the in vivo total CL based on a sensitivity analysis, which will be described later. The *PS_inf,act_* and *PS_diff_* were 2.5 L/h/kg and 0.8 L/h/kg, respectively, for the control group and 9.2 L/h/kg and 0.6 L/h/kg, respectively, for the repeated-dosing group (Table 3). The other mPBPK model parameters that were used are listed in Table 3.

**Figure 8:**
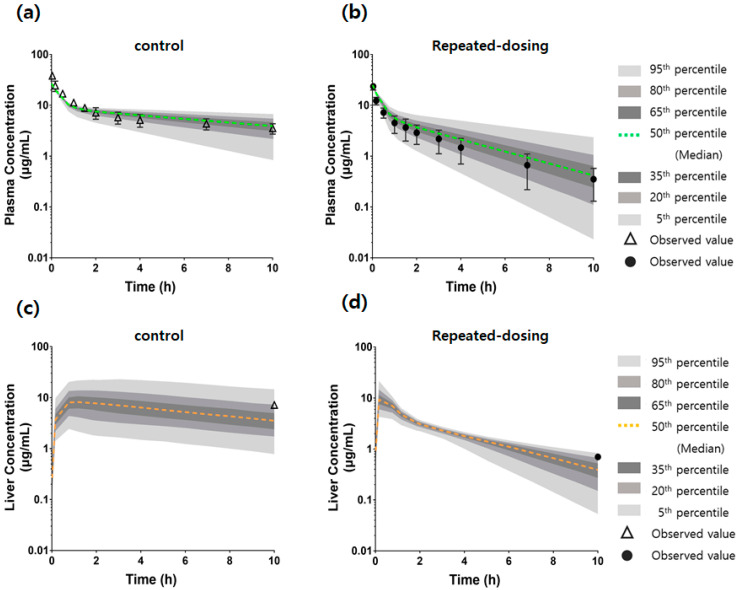
Simulated and observed intravenous plasma concentration–time profiles of SCR430 in the control and repeated-dosing groups. The Monte Carlo simulation curves (shaded region) and the observed data for the SCR430 concentrations in the plasma and liver of the control (open triangle; (**a**,**c**)) and repeated-dosing (closed circle; (**b**,**d**)) groups after intravenous administration at a dose of 3 mg kg^−1^. The shaded region represents 95% confidence intervals around the 5th and 95th (light gray-shaded region), 50th (green or yellow dotted line), 20th and 80th (middle gray-shaded region), and 35th and 65th (deep gray-shaded region) percentiles of the simulated concentration. The observed plasma concentrations are represented by means (symbols) and standard deviations (bars). The observed liver concentration is represented by a mean value.

The Monte Carlo simulation using the constructed mPBPK model showed a similar profile to the control and repeated-dosing groups’ observed plasma and liver concentrations. The observed concentration data in the plasma and liver for both of the groups were within the 95th percentile of the predicted data generated by the Monte Carlo simulations (Figure 8a–d). The prediction score with mean absolute percentage errors for the control and repeated-dosing groups showed an acceptable prediction (within 50%), and the determination coefficient (R^2^) was 0.85 for the control group and 0.93 for the repeated-dosing group. Especially, the time-dependent CL and liver concentration differences in the repeated-dosing group were reasonably predicted by the change in the *PS_inf,act_* and *PS_diff_* in the mPBPK model. The decreased liver concentration in the repeated-dosing group could be explained by the increased *f_u,hepa_* in the model.

### 3.8. Sensitivity Analysis

**Figure 9:**
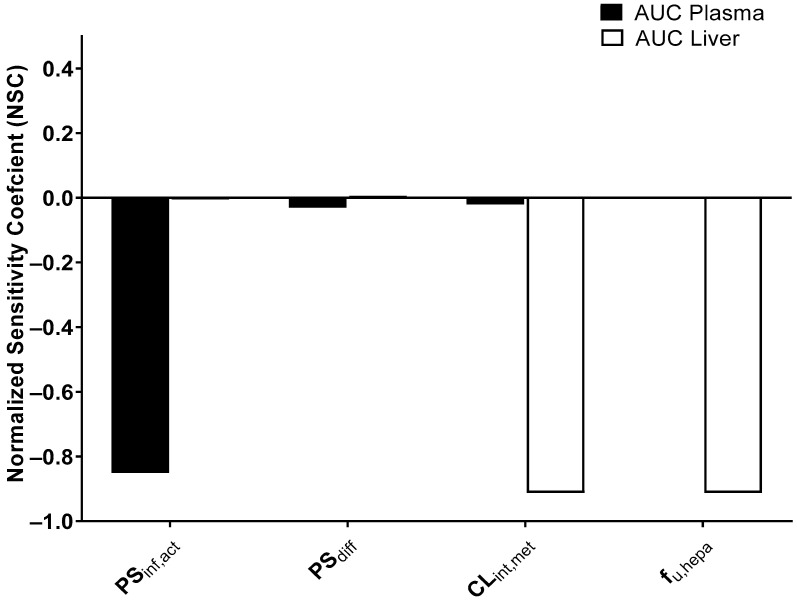
Local sensitivity analysis for the mPBPK modeling in the liver and plasma of the control group.

From all of the parameters related to hepatic elimination, *PS_inf,act_*, *PS_diff_*, *CL_int,met_*, and *f_u,hepa_* exhibited high absolute values of the normalized sensitivity coefficient (>0.2), and these parameters were used for a sensitivity analysis (Figure 9).

As expected from previous results, the plasma concentration was the most sensitive to the *PS_inf,act_* and less sensitive to the *PS_diff_*, *CL_int,met_*_,_ and *f_u,hepa_*. In contrast, the most influential factors for the liver concentration were *CL_int,met_* and *f_u,hepa_*.

### 3.9. The mRNA Expression Differences between the Three Groups

**Figure 10:**
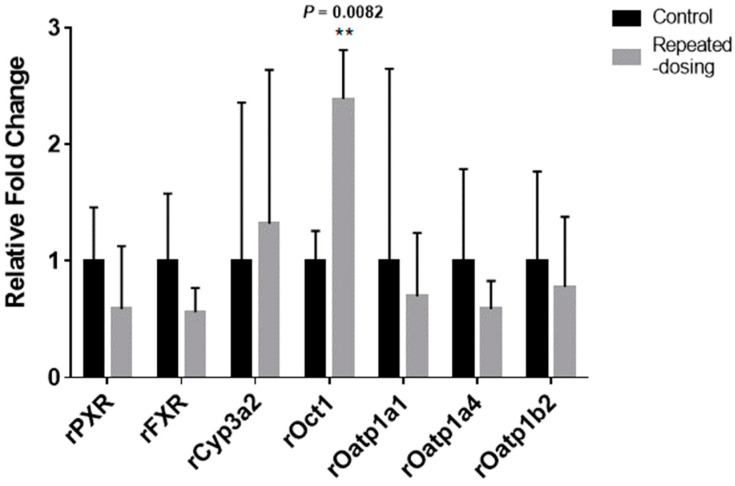
The relative expression of representative nuclear receptors, hepatic uptake transporters, and metabolic enzymes in the liver. The repeated-dosing group mRNA levels were expressed as a relative fold change compared to the control group (means ± the standard error of the mean). A Student’s *t*-test was employed to detect significant differences in the mRNA expression levels in the tissues (** *p* < 0.01).

The mRNA expression change, which is relevant for drug elimination in rat livers and kidneys, is summarized in Figure 10. The repeated-dosing group resulted in a significant increase in the expression levels of rOct1 in the liver (*p* = 0.0082). Additionally, the values of rPXR and rFXR tended to decrease, with no statistically significant differences.

## 4. Discussion

The auto-induction potential is a critical issue in the drug development stages and should be discussed for its quantitative impact on the PK parameters of the drug itself or co-administered drugs [45]. Until now, most auto-inductions were reported to be related to CYPs enzyme induction [46,47]. Only a limited number of studies are reported on hepatic transporter auto-induction along with metabolic enzyme auto-induction [48,49]. In this study, the underlying SCR430 TDPK mechanism in rats was investigated in terms of hepatic uptakes, metabolic activity, and binding in the matrix using IVIVE and PBPK modeling for the control and repeated-dosing groups.

The differences in the in vivo PK properties of the three groups following IV and IP administration at a 3 mg/kg dose were compared. The vehicle group did not alter the drug’s pharmacokinetic properties compared to the control group. However, a considerable decrease in the plasma concentration was shown in the repeated-dosing group compared to the control group (Figure 2a,b). The results indicate a time-dependent CL in the repeated-dosing group. Interestingly, the TDPK was more significant after the IP administration, which was thought to be due to the difference in the first-pass effect according to the administration route.

In contrast, the Vd_ss_ showed no significant differences in the groups (Table 1). The SCR430 elimination into the bile and urine as a parent form was negligible. The majority of the SCR430 was excreted into the bile in a hydroxylated metabolite form. This finding suggested that SCR430 appears to be cleared primarily by the liver and that the drug-induced induction in the hepatic elimination may be the primary SCR430 TDPK mechanism.

However, a SCR430 with a high molecular weight (≥400 Da), less likely biliary excretion, and high lipophilicity was considered cleared by the active transporter-mediated hepatic uptake process according to the extended clearance classification system [11,50]. Therefore, an ex vivo approach using isolated hepatocytes from the control and repeated-dosing groups was used in this study based on the extended clearance concept to discriminate the contribution of metabolic enzymes and hepatic transporters. The extended clearance concept is known to be useful in investigating the complicated interactions between metabolism and transport [10,11].

Interestingly, a statistically significant difference in the *CL_int,met_* was not observed between the three groups (Figure 5a–c), and the CL_h_ calculated using the IVIVE Method 1 (metabolic-dependent) could, therefore, not explain the time-dependent CL in the repeated-dosing group. However, the *PS_inf_* in the repeated-dosing group was larger than in the control group, and the CL_h_ calculated using the IVIVE Method 2 (uptake-dependent) could explain the SCR430’s time-dependent CL. Method 3 was expected to predict changes in the in vivo CL, similar to Method 2. Method 3 could partially predict the induction of the CL in the repeated-dosing group, but the prediction precision was lower than in Method 2. Therefore, the experimental observations in this study suggested that SCR430 may induce uptake transporters in rats’ livers, which could be the primary TDPK mechanism. The increased hepatic uptake may also explain the increased *Kp* of the kidney in the repeated-dosing group because hepatic uptake transporters, such as organic cation transporters, are also expressed in the kidney.

The mPBPK models for SCR430 also suggested that the inducible SCR430 hepatic uptake is most likely a contributor to the decreased plasma systemic exposure observed after multiple SCR430 dosing. The SCR430 plasma concentration–time profiles simulated by the mPBPK model, which incorporate parameters reflecting uptake induction, were in good agreement with the observed data in both of the groups (Figure 8). The Monte Carlo simulation and the sensitivity analysis results also demonstrated that the variations in the *PS_inf,act_* may be the primary explanations for the decrease in the systemic exposure following the repeated SCR430 administration. Moreover, the increased *f_u,hepa_* in the repeated-dosing group determined from in vitro hepatocyte assays could predict in vivo liver concentrations within a 2-fold range.

The mRNA expression level changes may indicate an increased *PS_inf,act_*. The rOct 1 expression showed about a 2.4-fold increase in the liver, whereas there were no significant changes in the metabolic enzyme’s mRNA expression. Interestingly, the nuclear receptors rPXR and rFXR were reported to suppress OCT1 expression [51,52]. The rPXE and rFXR expression levels showed a decreased tendency (Figure 10), although statistical significance was not found. However, the meaning of the mRNA expression results should be interpreted carefully because SCR430’s substrate specificity for rat transporters is not available and a limited number of genes were tested.

Unfortunately, we do not have a clear explanation for the change in the f_u,hepa,_ including *PS_diff_*, in the repeated-dosing group. However, the mechanism might be related to the SCR430 target considering that passive permeation through lipid membranes depends on lipid composition, and *f_u,hepa_* is known to depend on non-specific binding to the cellular phospholipids [53,54]. SHP-1 is already known to play a crucial role in lipid metabolism in the liver [55]. Further studies on the mechanism for changes in the *PS_inf,act_*, *PS_diff_*, and *f_u,hepa_* are likely needed.

As the chemical compound of interest has become more metabolically stable recently, transporter-mediated CL processes have become the primary factors behind clearance [25,56,57,58]. For instance, it has been demonstrated that the intrinsic hepatic uptake CL in the case of statins, which was determined in human hepatocyte suspension, was far more accurate for predicting the in vivo clearance than extrapolating directly from the intrinsic metabolic CL [16,59]. Therefore, our results, TDPK by auto-induction of the hepatic uptake process, and approach, mechanistic analysis using IVIVE and PBPK, are worth carefully considering in new drug development processes.

## 5. Conclusions

This study’s results revealed that SCR430 showed time-dependent CL after repeated doses and was related to the hepatic uptake process auto-induction, not to metabolic activity. This is the first report on TDPK induced by the transport process without metabolic involvement.

The ex vivo approach combined with IVIVE and PBPK, established in our study, could help mechanistically clarify complex auto-induction mechanisms in the drug development stage.

## Figures and Tables

**Table 1:** Comparison of the pharmacokinetic parameters between the control (without a dosing vehicle or SCR430, *n* = 6), vehicle (vehicle only, 14 days, PO, *n* = 6), and repeated-dosing (SCR430 30 mpk, 14 days, PO, *n* = 6) groups. The data are presented as mean ± standard deviation (relative standard error %).

Parameter	Control Group	Vehicle Group	Repeated-Dosing Group
IV (3 mg/kg)			
AUC_0→∞_ (μg/mL·min)	6333 ± 1505 (9.7%)	6319 ± 2854 (18.4%)	1420 ± 446 ** (12.8%)
CL (mL/h/kg)	30.02 ± 8.29 (11.3%)	32.99 ± 12.54 (15.4%)	137.9 ± 43.8 *** (12.9%)
Vd_ss_ (mL/kg)	286.4 ± 65 (9.2%)	316.0 ± 58.7 (7.6%)	378.4 ± 119.3 (12.8%)
CL_b_ (mL/h/kg)	0.05 ± 0.02 (22%)	0.061 ± 0.01 (9.8%)	0.075 ± 0.003 (1.3%)
CL_r_ (mL/h/kg)	0.005 ± 0.002 (20%)	0.003 ± 0.002 (33%)	0.007 ± 0.006 (42%)
*R_b_*	3.95 ± 0.64 (9.4%)	4.58 ± 0.47 (5.9%)	4.53 ± 0.65 (8.2%)
IP (3 mg/kg)			
AUC_IP,0→10h_ (ng/mL·h)	7604 ± 3110 (16.7%)	6894 ± 1004 (5.9%)	951 ± 302 *** (12.9%)
Binding properties			
Fraction unbound (*f_u_*)			
*f_u,__p_* ^a^	0.0078 ± 0.001 (7.7%)	0.0074 ± 0.002 (13.5%)	0.0081 ± 0.002 (12.3%)
*f_u__,inc,hepa_* ^b^	0.032 ± 0.004 (6.2%)		Equal to control
Predicted *f_u__,inc,heap_* ^c^	0.024		0.024
Extrapolated *f_u__,inc,hepa_* ^d^	0.088		0.088
*f_u,hepa_* ^e^	0.004 ± 0.002 (25%)		0.01 ± 0.008 (40%)
Tissue:Plasma Partition Coefficients (*Kp*)			
*Kp_liver*	2.06 ± 0.06 (1.4%)	1.27 ± 0.15 (6.3%)	1.96 ± 1.09 (32.1%)
*Kp_kidney*	1.20 ± 0.1 (5%)	1.37 ± 0.15 (5.8%)	5.50 ± 0.82 * (8.5%)

^a^ determined experimentally. ^b^ determined experimentally. ^c^ predicted by an in silico method [39]. ^d^ extrapolated from plasma protein binding [40]. ^e^ determined by a *Kp* method at 4 °C in 120 min. * *p* < 0.05, ** *p* < 0.01, *** *p* < 0.001.

**Table 2:** Predicted and observed hepatic clearance calculated using IVIVE Methods 1, 2, and 3.

	Unit	Control	Vehicle	Repeated-Dosing
Method 1				
in vitro intrinsic metabolic clearance (*CL_int,met,vitro_*)	μL/min per 10^6^	1.32	1.21	1.69
in vivo overall intrinsic clearance (*CL_int,all_*)	mL/min per kg	160.4	147.0	205.3
Predicted hepatic clearance (CL_h_)	mL/h per kg	74.8	68.6	95.7
Method 2				
in vitro hepatocyte uptake clearance (*PS_inf,hep_*)	μL/min per 10^6^	11.9		43.2
in vivo overall intrinsic clearance (*CL_int,all_*)	mL/min per kg	46.2		168.0
Predicted hepatic clearance (CL_h_)	mL/h per kg	21.6		78.4
Method 3				
intrinsic metabolic clearance (*CL_int,met_*)	mL/min per kg	160.4		205.3
liver-to-plasma unbound drug concentration ratio at steady state (*Kp_uu,ss_*)		0.11		1.7
in vivo overall intrinsic clearance (*CL_int,all_*)	mL/min per kg	17.3		344.3
Predicted hepatic clearance (CL_h_)	mL/h per kg	8.1		160
Observed in vivo clearance (CL)	mL/h per kg	30.02	32.99	137.9

**Table 3:** Input parameter values used for the mPBPK modeling and Monte Carlo simulation.

Parameters			Comment
	Control	Repeated-Dosing	
Single-adjustment compartment			
k_in_ (hr^−1^)	2.7	Equal to Control	Fitted from observed data
k_out_ (hr^−1^)	1.0	Equal to Control	Fitted from observed data
Tissue volume (L/kg)			
Central	0.018	Equal to Control	Fitted from observed data
Liver	0.037	Equal to Control	[28]
Extracellular space in the liver	0.01	Equal to Control	[44]
Hepatocytes	0.027	Equal to Control	[44]
Blood flow (L/h/kg)			
Liver	3.69	Equal to Control	[28]
Monte Carlo simulation parameters			
*PS_inf,act_* (L/h/kg)	(2.5, 0.8)	(9.2, 0.6)	(Mean, SD)
*PS_diff_* (L/h/kg)	(0.3, 0.09)	(0.86, 0.4)	(Mean, SD)
*CL_int,met_* (L/h/kg)	(9.8, 1.0)	(12.3, 0.08)	(Mean, SD)
*f_u,hepa_*	(0.004, 0.002)	(0.01, 0.008)	(Mean, SD)

## Appendix A

### Appendix A.1. Analytical Method

All of the samples were processed with liquid–liquid extractions. Aliquots (50 μL) from each sample were transferred into 2 mL microfuge tubes, and the reaction was terminated by adding 1 mL of ice-cold methyl tert-butyl ether solution and 50 μL sorafenib, an internal standard. After vortex mixing, followed by centrifugation at 7000× *g* for 10 min, 900 μL of the supernatants was evaporated by drying under a stream of nitrogen gas. The residues were reconstituted with 100 μL of the mobile phase for the LC-MS/MS analysis. An Acquity UPLC (Waters, Milford, MA, USA) with a Waters Quattro micro™ API mass spectrometer (Waters, Milford, MA, USA) and a Meteoric Core C18 100A, 100 × 2.0 mm, 2.7 µm (YMC, Kyoto, Japan) were used for the study.

An isocratic mobile phase consisting of an 80:20 mixture of 0.01% formic acid in acetonitrile (ACN) and 0.01% formic acid in distilled water was delivered at a flow rate of 0.2 mL/min. Quantification was achieved using electrospray ionization-MS/MS detection in the positive ion mode for the SCR430, its oxidized metabolite, and sorafenib. The optimal instrumental parameters were as follows: capillary voltage of 3.5 kV, cone voltage of 40 V, source temperature of 100 °C, and desolvation temperature of 300 °C. Nitrogen was used as the desolvation gas and the cone gas, with flow rates of 500 and 50 L/h, respectively. The collision energy was 20 V. Detection of the ions was carried out in the multiple reaction-monitoring mode, and the settings are shown in Appendix B, Table A3. The identification of the SCR430 metabolites in the plasma, liver tissue, bile, and hepatocytes was achieved using UPLC-quadrupole time-of-flight MS (qToF-MS), SYNAPT G2-Si HDMS, (Waters, Milford, MA, USA). The settings of the qToF-MS were as follows: MS scan range, 60–1400 *m*/*z* (ESI (-) mode, capillary voltage of 2 kV, cone voltage of 10 V, source temperature of 100 °C, and desolvation temperature of 350 °C.

### Appendix A.2. The Mass-Balanced Differential Equation for the Liver Compartments

A permeability-limited model was used for each compartment, incorporating the active transporter-mediated uptake (*PS_inf,act_*), the passive diffusion clearance (*PS_diff_*), and the intrinsic metabolic clearance *CL_int,met_*. The corresponding differential equations referring to the liver were as follows:

Extracellular compartment 1:

(A1) $$\frac{V_{he}}{5}\times \frac{dC_{he,1}}{dt}=Q_{h}\times \left(C_{sys}-C_{he,1}\right)-\frac{PS_{inf,\;act}+PS_{\;diff}}{5}\times f_{u,\;b}\times C_{he,1}+\frac{PS_{diff}}{5}\times f_{u,\;hepa}\times C_{hc,1}\;$$

Extracellular compartment 2~5 (i = 2~5):

(A2) $$\frac{V_{he}}{5}\times \frac{dC_{he,i}}{dt}=Q_{h}\times (C_{he,i-1}-C_{he,i})-\frac{PS_{inf,\;act}+PS_{\;diff}}{5}\times f_{u,\;b}\times C_{he,i}+\frac{PS_{diff}}{5}\times f_{u,\;hepa}\times C_{hc,i}\;$$

Hepatocellular compartment 1~5 (i = 1~5):

(A3) $$\frac{V_{hc}}{5}\times \frac{dC_{hc,i}}{dt}=\frac{PS_{inf,\;act}+PS_{diff}}{5}\times f_{u,b}\times C_{he,i}-\frac{PS_{diff}+CL_{int,met}}{5}\times f_{u,hepa}\times C_{hc,i}$$

where *C_he_*, *C_hc_*, *C_sys_*, and *V_he_* are the concentration in the liver extracellular compartment, the concentration in the hepatocellular compartment, the concentration in the systemic compartment, and the volumes of the extracellular compartments, respectively (Table 3).

## Appendix B

**Table A1:** Primer sequences and PCR conditions used for the qPCR.

Gene	Forward Primer (5′-3′)	Reverse Primer (5′-3′)	Product Size (bp)	PCR Conditions
rGapdh	AACGACCCCTTCATTGAC	TCCACGACATACTCAGCAC	191	D: 95 °C for 10 s, A: 57 °C for 30 s, E: 72 °C for 30 s
rPXR	TCCACTGCATGCTGAAGAAG	AACCTGTGTGCAGGATAGGG	187	D: 95 °C for 10 s, A: 56 °C for 30 s, E: 72 °C for 40 s
rFXR	CCAACCTGGGTTTCTACCC	CACACAGCTCATCCCCTTT	183	D: 95 °C for 10 s, A: 56 °C for 30 s, E: 72 °C for 40 s
rCyp3a2	CGACTTGGAACCCATAGAC	CATGTCAAATCTCCCTAAGCC	117	D: 95 °C for 10 s, A: 58 °C for 30 s, E: 72 °C for 30 s
rOatp1a1 (Oatp1)	ACCTGGAACAGCAGTATGGAAAA	ACCGATAGGCAAAATGCTAGGTAT	163	D: 95 °C-5 to 10 s, A: 56 °C for 6 s, E: 72 °C for 6 s
rOatp1a4 (Oatp2)	TGTGATGACCGTTGATAATTTTCCA	TTCTCCACATATAGTTGGTGCTGAA	81	D: 95 °C-5 to 10 s, A: 56 °C for 10 s, E: 72 °C for 6 s
rOct1	CTCTGGCTACAGGAGAACGAC	CCTGGTACAGCACAGCACAA	401	D: 94 °C for 30 s, A: 60 °C for 30 s, E: 72 °C for 30 s

D: denaturation, A: annealing, E: elongation.

**Table A2:** Drug physicochemical properties.

Parameter	Value
Physicochemical properties	
Molecular weight (g/mol)	431.8
pKa	6.27 ^a^
clogD7.4	5.95 ^b^
clogP	6.15 ^b^

^a^ Calculated by a machine learning-based model for pKa prediction [60]. ^b^ Predicted by the ACD/LogP program.

**Table A3:** Summary of monitoring ions and respective parameters for MS detection.

Name	Q1 Mass	Q3 Mass	Dwell Time (s)	Cone (V)	Collision (V)
SCR430	432.1	196.1	0.15	40	20
Hydroxylated metabolite	448.1	212.1	0.15	55	25
Sorafenib (IS)	464.9	252.2	0.15	55	38
